# Synthetic Data Generation for Automatic Segmentation of X-ray Computed Tomography Reconstructions of Complex Microstructures

**DOI:** 10.3390/jimaging9020022

**Published:** 2023-01-19

**Authors:** Athanasios Tsamos, Sergei Evsevleev, Rita Fioresi, Francesco Faglioni, Giovanni Bruno

**Affiliations:** 1 Bundesanstalt für Materialforschung und-Prüfung (Federal Institute for Materials Research and Testing), 12205 Berlin, Germany; 2 Department of Farmacy and Biotechnology (FABIT), University of Bologna, 40126 Bologna, Italy; 3 Department of Chemical end Geological Sciences (DSCG), University of Modena and Reggio Emilia, 41125 Modena, Italy; 4 Institute of Physics and Astronomy, University of Potsdam, 14476 Potsdam, Germany

**Keywords:** automatic segmentation, 3D deep convolutional neural network (3D DCNN), Dice score, metal matrix composite (MMC), modified U-Net architectures, multi-phase materials

## Abstract

The greatest challenge when using deep convolutional neural networks (DCNNs) for automatic segmentation of microstructural X-ray computed tomography (XCT) data is the acquisition of sufficient and relevant data to train the working network. Traditionally, these have been attained by manually annotating a few slices for 2D DCNNs. However, complex multiphase microstructures would presumably be better segmented with 3D networks. However, manual segmentation labeling for 3D problems is prohibitive. In this work, we introduce a method for generating synthetic XCT data for a challenging six-phase Al–Si alloy composite reinforced with ceramic fibers and particles. Moreover, we propose certain data augmentations (brightness, contrast, noise, and blur), a special in-house designed deep convolutional neural network (Triple UNet), and a multi-view forwarding strategy to promote generalized learning from synthetic data and therefore achieve successful segmentations. We obtain an overall Dice score of 0.77. Lastly, we prove the detrimental effects of artifacts in the XCT data on achieving accurate segmentations when synthetic data are employed for training the DCNNs. The methods presented in this work are applicable to other materials and imaging techniques as well. Successful segmentation coupled with neural networks trained with synthetic data will accelerate scientific output.

## 1. Introduction

### 1.1. Related Work

In recent years there has been an upsurge in the use of artificial intelligence (AI) in material science-related problems. More specifically, the number of studies employing artificial deep convolutional neural networks (DCNNs) for the qualitative and quantitative analysis of X-ray computed tomography (XCT), magnetic resonance imaging (MRI), and microscopy data has continuously been increasing. This technology was originally developed for the automatic analysis and segmentation of various biological organs, cells, tumors, etc., from biomedical XCT and microscopy data [1,2,3,4]. It did not take long for this method to expand to material science as well. For instance, in [5], 2D DCNNs were employed for the segmentation and classification of microstructural constituents present within low-carbon steel from images retrieved with scanning electron microscopy (SEM) and light optical microscopy (LOM). In [6], 3D DCNNs were used for the semantic segmentation of short glass fibers within a short-fiber-reinforced polymer (SFRP) from XCT scans. Automatic segmentation of defects was successfully performed on XCT data of additively manufactured materials with a 3D DCNN [7] and conventional materials with a 2D DCNN [8]. A similar study was conducted by Strohmann et al. [9]: micro-XCT scans of the microstructure of a three-phase Al–Si alloy were semantically segmented (phases: aluminum alloy matrix, silicon, and aluminide intermetallics) with a state-of-the-art 2D DCNN. Excellent segmentation performance was reported based on manually labeled training data coupled with a pixel-wise weighted loss function [10] for the working net. Another important study [11] was carried out by Evsevleev et al., which is relevant for this work because of the large number of phases present: XCT scans of a five-phase AlSi12CuMgNi metal matrix composite (MMC) alloy reinforced with short Al_2_O_3_ fibers and SiC particles were semantically segmented with a 2D DCNN, despite the major challenges (lack of contrast among phases) involved. As in many other studies, manually annotated semantic labels were used as training data for the working neural network. For 2D networks, the training data required to obtain accurate results are not vast. Generally, only a few slices (cross-sections) are required to be manually segmented and labeled to thoroughly train a 2D DCNN [9,12]. If data augmentations are adopted [11], the required number of labeled data can further be reduced. This is indeed the aim of our work, i.e., to provide a method for data augmentation for effective neural network training.

### 1.2. Problem and Motivation

A lot of multiphase composite materials possess complex microstructures, both because of the different constituents and because of diverse geometrical features such as the constituents’ shape, size, orientation, etc. A compelling example of such materials is Al–Si MMCs with multiple reinforcing phases. For such materials, the exact mechanical and fracture properties have not been extensively studied. Arguably, the best tool for the visualization and analysis of their microstructure is XCT due to its 3D nature. However, many challenges exist. It is not uncommon for certain individual microstructural phases to share similar X-ray attenuation coefficients (similar densities). This consequently leads to similar grayscales in reconstructed XCT data. Therefore, both simplistic thresholding [13] and even the more sophisticated Otsu thresholding algorithm [14] are rendered ineffective as segmentation methods. On the other hand, complete manual segmentation is impractical due to the enormous effort and time required. Finally, even with an artificial intelligence-based (AI) approach, this problem can still be challenging as training data must be manually annotated. Nevertheless, in [9], it was reported that the total time required to fully segment XCT data with AI was less than 1% compared to a manual approach. However, this did not include the time needed to manually annotate the required training data. As mentioned previously, for 2D networks, the training data requirements are not enormous, especially when the microstructure contains only a limited number of phases. Furthermore, if many microstructural phases are present, geometrical features become more and more important for accurate feature recognition. For instance, in [11], it is reported that due to the similar grayscales of the reinforcing SiC particles and Al_2_O_3_ fibers, the in-plane vertical fibers were wrongly classified as particles with a 2D DCNN. Thus, a 3D network could have potentially performed better. For 3D DCNNs, the limitation is the vast number of required training data. Some researchers have overcome similar problems by averaging resulting output segmentation probabilities from multiple plane views with 2D networks [15,16]. Nonetheless, manually annotated labels are still required for training, with the number of slices needed depending on the complexity of the problem to be tackled. Reasonably, some researchers have attempted to train the working neural networks with synthetic or simulated data [6], apart from the manually annotated training data. The results revealed very close segmentation accuracies between the two approaches. However, Konopczyński et al. [6] studied a rather straightforward case with only two phases (polymer matrix and glass fibers), possessing very distinct grayscales. Similarly, in [17], 2D synthetic data were employed to segment vanadium pentoxide (V_2_O_5_) nanowires in transmission X-ray microscopy (STXM) and scanning electron microscopy (SEM) 2D images. As before, the problem involved only two phases (V_2_O_5_ wires and background) with very distinct grayscales, hence a simpler classification task. Other more recent studies incorporating synthetic data training are [18] and [19], in which grain boundaries in polycrystalline iron and defects (cracks, voids) in steel workpieces, respectively, were segmented with DCNNs trained with synthetic data. A segmentation study of a multiphase composite material with a complex microstructure and similar phase grayscales, such as the Al–Si MMC presented in [11], still remains a major challenge. In this work, we lay the path to the solution to the problem of a reproducible and precise segmentation of XCT data. A successful segmentation of XCT data on this material with a 3D DCNN trained with synthetic data will certainly enable reliable quantitative microstructural analysis (phase fractions, shape, distribution of phases, etc.). Such a segmentation strategy could be applied to many other equally challenging microstructures. Furthermore, a segmentation approach based on synthetic microstructures does not necessarily have to be limited to XCT data but could reasonably be applied to other imaging techniques, such as microscopy, neutron diffraction, ultrasonics, etc. Additionally, minimum human intervention (no manual labeling) will accelerate scientific output, reduce potential human error, and further solidify AI applications in materials research. Lastly, in [5], it is reported that segmentation performance on smaller objects with the employed neural network was poor. From our experience, this is not an uncommon occurrence even with current state-of-the-art neural network architectures (e.g., accurate segmentation of thin fibers, few pixels/voxels thick). We will show that our novel architecture overcomes this limitation.

### 1.3. Research Approach Outline

The research steps undertaken are outlined below:

1. An in-house MATLAB library was coded and used to model/simulate synthetic Al–Si MMCs microstructures based on the MMC reported in [11]. Such synthetic microstructures were generated to appear similar to those of an XCT scan in terms of both structural resemblance and simulated grayscales.
2. The synthetic microstructures were then sliced and augmented, as detailed below, to provide suitable training data for an in-house designed 3D UNet [4,20] DCNN architecture.
3. After training, the suggested architectures were coupled with several forwarding strategies to segment the XCT experimental data. The term, forwarding strategy, refers to the slicing method of the data into smaller batches, the subsequent passage of these batches through the working networks, and finally, their recombination into the final semantically segmented XCT data reconstructed volumes.
4. The performance was assessed based on the Dice precision coefficient, a commonly used segmentation performance metric of DCNNs. The precision was assessed both on a synthetic XCT volume (used only for testing) and on experimental XCT volumes from which arbitrary slices were extracted and manually labeled as the ground-truth benchmark.

## 2. Material Description

**Figure 1:**
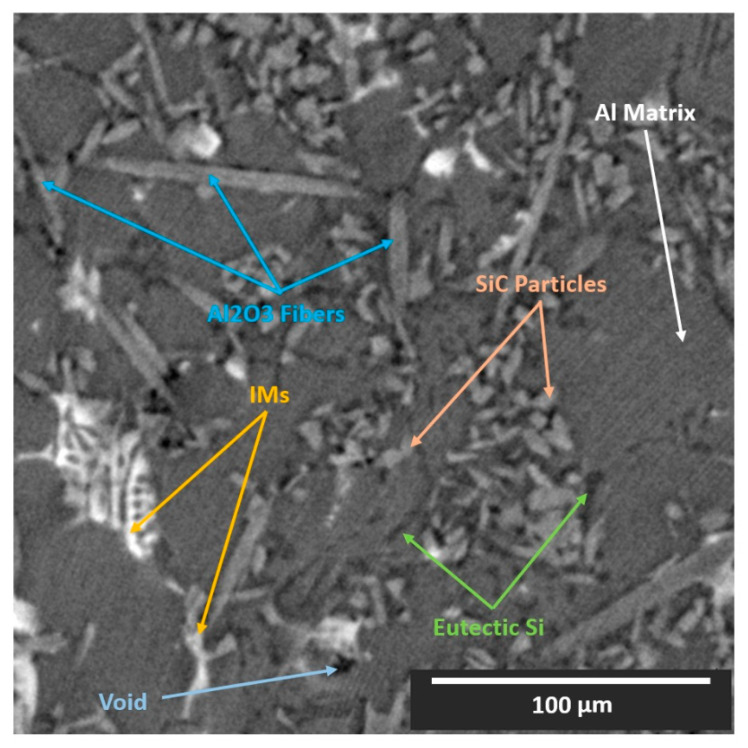
XCT reconstruction slice (XY-plane view) of the AlSi12CuMgNi MMC.

Cast near-eutectic Al–Si alloys are some of the most common materials currently employed in the automotive industry for engine piston production [21]. Furthermore, there has been an increased interest in the exploitation of these materials in the aerospace industry, aiming to substitute the broadly used unreinforced Al and Ti alloys [22]. Examples include aerials, frames, and joining elements. The addition of the Si phase introduces high fluidity to the melt. Moreover, transition elements such as Cu, Fe, and Ni promote the formation of stable aluminides (intermetallics), which along with the eutectic Si phase, assemble a 3D interconnected network within the microstructure of the alloy [23]. To improve certain mechanical properties, such as creep resistance and strength, MMCs of these materials are formulated with the addition of reinforcing phases such as ceramic particles and/or short ceramic fibers [11,21,22,23]. This is to suppress the over-aging deterioration of the aluminum matrix, occurring at extended service at high temperatures, even >300 °C. Thus, the microstructure of such composites typically consists of four or five phases (Al matrix, eutectic Si, intermetallics (IMs), ceramic short fibers, and ceramic particles), or even six phases, if voids and cracks are considered. The complex 3D microstructural nature of the composites justifies the extensive use of XCT for their microstructural analysis. The specific composition of the material examined in this study is an AlSi12CuMgNi alloy reinforced with 7%vol Al_2_O_3_ short fibers and 15%vol SiC particles produced by squeeze casting. The hybrid preform, in which the molten alloy was infiltrated, had a priori the reinforcing fibers planar-randomly orientated (XY-plane) and the reinforcing particles randomly distributed. Synchrotron XCT (SXCT) data were acquired at the BAM*line* beamline at the BESSY II synchrotron in Berlin, Germany. A monochromatic beam with an energy of 25 keV was used; the pco camera allowed an effective pixel size of (0.44 μm)^2^; 2400 projections were acquired with a counting time of 3 s/projection; and the reconstruction of the raw data was made using Paganin’s phase retrieval method and a filtered back-projection algorithm. The exact experimental procedure and equipment used for the SXCT imaging and the analysis of the microstructure are described in detail in [11]. A 512 × 512 (pixels) cross-section of the XCT reconstruction of the material is shown in Figure 1. We observe that certain specific phases (particles, fibers, Al matrix, and some intermetallics) share similar X-ray attenuation coefficients.

## 3. Method Development

### 3.1. Synthetic Al–Si MMC Microstructure Generation

#### 3.1.1. General Strategy

In the next paragraph, we outline the approach; details of each step will be given in the following sections.

The in-house developed MATLAB library (BAM SynthMAT, MathWorks, Natick, MA, USA) can model a variety of microstructures, including particles and fibers, in terms of shape (convex/concave, roundness), aspect ratio, size, etc. These can be positioned within a user-defined individual volume with various positioning functions, thereby controlling the orientation and statistical positioning (i.e., Monte Carlo, nearest neighbor distance). If required, non-overlapping (hardcore) positioning can be imposed within the individual single-phase volume during its formulation process (internally self-dependent hardcore positioning) or in conjunction with other already formulated individual volumes occupying the same space (externally dependent hardcore positioning), or both. The final assembly into a global final volume is accomplished by combining all the formulated individual volumes with a priority function. A user-defined priority number is assigned to each individual volume. The voxels of certain individual single-phase volumes with higher priority can cover the voxels of other individual volumes/phases with lower priority during the final assembly if they occupy the same space. Finally, functions have been coded to assign grayscales as required and simulate local phase contrast, noise (Poisson, Gaussian), and blur (Gaussian, mean). The resulting synthetic volumes are extracted/saved as raw 8-bit binary data with assigned grayscales (range: 0–255) and as raw 8-bit binary data containing specified labels for the various synthetic phases (range: 0, 1, …, [no. of phases—1]). More specifically, in the case of the Al–Si MMC, there are 6 different phases present: Al_2_O_3_ fibers, SiC particles, IMs, eutectic Si, voids, and Al matrix. Thus, a few important aspects were considered before building the synthetic microstructures. Simple observations within the experimental XCT data were sufficient for this task. We are going to elucidate them, together with our effective procedure for the generation of synthetic microstructures, in our next section.

#### 3.1.2. Synthesis Preprocessing—Required Information

First, the interactions between the phases must be defined. For instance, fibers can interpenetrate all the phases apart from the ceramic particles. Self-dependent hardcore positioning is an obvious requirement for this phase. Similar principles apply to the ceramic particles and the voids phases as well. On the other hand, the IMs and eutectic Si phases have lower priorities. Therefore, fibers, particles, and Voids can exist within the latter. However, no eutectic Si can be enclosed within the IM phase. The Al matrix phase has the lowest priority of all phases.

Secondly, the geometrical parameters had to be recorded for each individual phase. For the fibers, the approximate length/thickness range, approximate orientation/inclination (mostly coplanar with XY-plane, randomly oriented), and distribution were taken into account, and similarly, for the particles, the approximate aspect ratio, size, and roundness (convexity) were considered. For instance, particles and voids have approximately an aspect ratio between 1 and 2, with a rather round or polygonal shape and random orientation. The eutectic Si phase resembles a network consisting of relatively thin platelet-shaped interconnected particles [24,25], with an aspect ratio approximately between 1 and 3, and a random orientation and distribution. The IMs phase is quite concave in nature, with a structure resembling dendrite and/or cave-like formations, random orientation, and distribution.

**Figure 2:**
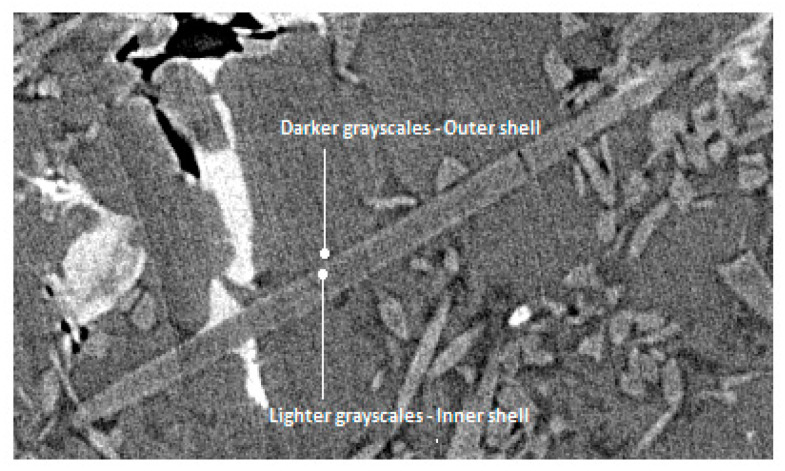
Local phase contrast at an Al_2_O_3_ fiber/Al matrix interface.

Finally, grayscales of individual phases were sampled from various locations within the experimental XCT data. A conservative strategy is to generate several average values for each individual phase and not just a global average. More specifically, each individual average value should be calculated by averaging samples from different positions (voxels) within a single object (e.g., the same fiber, the same particle, the same IM, etc.) and not across the whole XCT reconstructed volume. The next individual average value for the same phase can be the average grayscales from a similar object located elsewhere. Furthermore, there can be interfaces where local fluctuations in grayscales are present due to phase contrast effects. In such cases, it is beneficial to note down the increased/decreased grayscale values in that region. For instance, this can be observed mostly around thick fibers, where inner brighter and outer darker grayscales emerge at the interface in a double shell formation (Figure 2). The sampling of the geometrical parameters and the grayscale values does not have to be an extremely precise process. The training strategies shown below would anyway refine the identification process. The meticulous recording of how the involved individual phases merge/interact with each other (and with themselves) is nevertheless imperative. These properties control the assembly hierarchy of the individual phases during the formulation process of the synthetic microstructures.

#### 3.1.3. The Synthesis Process

The generation of the synthetic Al–Si MMC microstructures is divided into four major sequential steps:

1. Fabrication and enrichment of individual particle and fiber repositories.
2. Positioning of individual particles within individual single-phase volumes with the various positioning functions.
3. Final synthetic volume assembly (by merging individual single-phase volumes with a priority function).
4. Assignment of grayscales and local phase contrast (where applicable).

#### 3.1.4. Individual Particles and Fibers Fabrication

**Figure 3:**
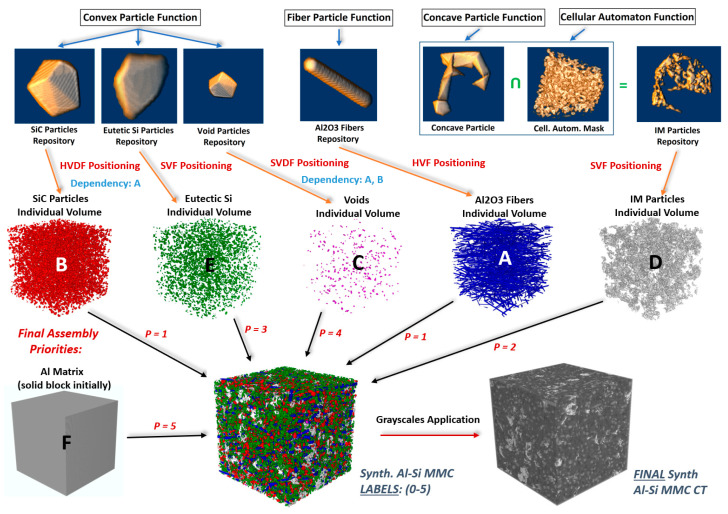
Synthetic Al–Si MMC generation procedure flowchart.

• Fiber Particle Function (Al_2_O_3_ Fibers): For the individual fibers, the only parameters required are length and diameter (both in terms of the number of voxels). With a coded function, we designate in a prismatic volume (size: fiber’s length x diameter x diameter), which voxels occupy the fiber space (i.e., 0: empty, 1: fiber). Such data are saved in binary form. The information required to properly load the geometry (size: x, y, z) can be stored in the initial bits of the file in the form of a header with a fixed length. The file name can have an integrated increasing integer serial number, representing the total number of fabricated fibers in a repository.

• Convex Particle Function (SiC, Eutectic Si, and Void particles): For the individual convex particles, a convex hull function can be used [26], which can generate all the mathematically possible convex hulls based on a number of points. This can randomly be assigned between a specific user-defined range. Moreover, the points can lie within a prism of user-defined dimensions, effectively controlling size and aspect ratio. The number of points controls the particle roundness. In a similar manner to the individual fibers, the data can be voxelated and saved as binary data (the same principles apply).

• Concave Particles and Cellular Automaton Functions (IMs particles): For the individual IMs particles, essentially the same procedure as above is followed. However, as concave particles are required here, an alpha radius parameter is introduced [27], which effectively controls the concavity of the IM particles. This parameter can be viewed as the diameter of a small sphere. If the latter can fit through any triangular face of a generated convex hull, then this hull is excluded/deleted. Thus, the smaller the Alpha-Radius is, the higher the concavity of the final particle. The dendrite and cave-like structures were introduced with the intersection of the generated concave particle with a cellular automaton devised 3D mask resembling cave structures (see Figure 3). The 3D cellular automaton rule was inspired by the Game of Life paradigm [28].

#### 3.1.5. Positioning Functions

Independently from the function used for data generation, particles/fibers were loaded from the formulated repositories, resized (if applicable, as required), and rotated (if applicable, as required) before being positioned into the individual single-phase volumes. The coded positioning functions that were used for the synthesis of individual volumes are detailed below.

• Simple Volume Function (SVF): Particles from the repositories are randomly positioned and rotated as required until a (user-defined) volume fraction is achieved (particles can overlap). Applications were IM and eutectic Si phases.

• Hardcore Volume Function (HVF): Particles from the repositories are randomly positioned and rotated as required until a (user-defined) volume fraction is achieved with a self-hardcore requirement (particles cannot overlap). Application was Al_2_O_3_ fibers phase.

• Simple Volume-Dependent Function (SVDF): Particles from the repositories are randomly positioned and rotated as required until a (user-defined) volume fraction is achieved with an external hardcore requirement (particles cannot overlap other particles from an external volume occupying the same space, but they can self-overlap). Application was the voids phase.

• Hardcore Volume-Dependent Function (HVDF): Particles from the repositories are randomly positioned and rotated as required until a (user-defined) volume fraction is achieved, with both internal and external hardcore requirements. Application was the SiC particles phase.

#### 3.1.6. Generated Synthetic Volumes

**Figure 4:**
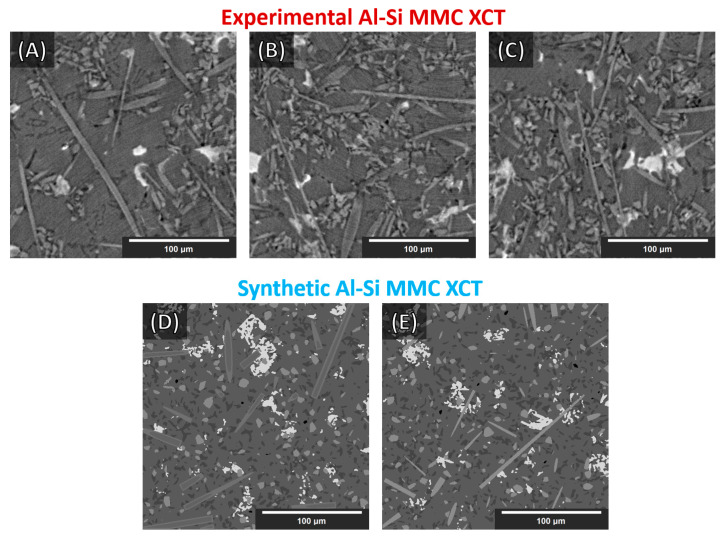
Slices (XY-plane view) of reconstructed XCT data of Al–Si MMCs (**A**–**C**) and synthetic Al–Si MMC (**D**,**E**) sample microstructures. (The augmentations on the synthetic data were not applied directly on the whole synthetic volumes. See Section 3.2.2.)

Eight 512 × 512 × 512 synthetic Al–Si MMCs microstructures were generated with various parameters (different volume fractions, particles, and fibers sizes/lengths, orientations, grayscales, etc.). Statistics and parameters are included in Appendix A (Table A1, Table A2 and Table A3). Some cross-sections of these structures are illustrated in Figure 4 (Figure 4D,E). Finally, the whole synthesis procedure is summarized as a flowchart in Figure 3 (the top line represents the single objects created according to the procedure outlined in Section 3.1.4).

### 3.2. Training Data

#### 3.2.1. Augmentations on Training Data

From the eight synthetic Al–Si MMC volumes formulated, seven were randomly selected for generating the training and validation data for the neural networks. The remaining volume was kept as testing data for the final performance assessment. Independently from the synthesis of the artificial XCT data for training, the experimental grayscale distributions cannot be perfectly replicated. In fact, the synthetic data incorporated only a few grayscales to represent the various microstructural phases. Therefore, a strategy was devised to ensure generalization during training. Traditionally, researchers have been using image augmentations (resizing, rotations, distortions, etc.) to increase the number of training data available when numbers are not sufficient. In our approach, we employ only four specific augmentations, aiming to render the neural networks more versatile in segmenting experimental data (with knowledge gained from synthetic data). More specifically, we applied contrast and brightness augmentations, in random orders and intensity (+/− 10% for both). We also added moderate Gaussian noise (random standard deviation = 0–8 for a grayscale range: 0–255) and 3D Gaussian spatial blur (random sigma = 0–1) in both the training and validation datasets. The generalization expected from the brightness/contrast augmentations was the ability of the network(s) to comprehend different material interfaces in experimental datasets based on synthetic training datasets. This forced the DCNN to consider 3D interface geometry as well (particle shapes and interface grayscale differences). The purpose of the added noise and blur was to render the training a bit more challenging and, thus, further improve generalization. Lastly, experimental XCT data show regions with densely packed particles and regions where particles are loosely scattered. Such different arrangements were separately reproduced and then combined in the final synthetic microstructure, which therefore contained inhomogeneous particle spatial distribution and volume fractions. This feature enhanced the DCNN’s ability to accurately semantically segment particles both tightly packed and loosely clustered (e.g., to avoid tightly clustered SiC particles or Al_2_O_3_ fibers being mislabeled as the Al-matrix phase and vice versa). Therefore, a statistical phase distribution generalization was introduced.

#### 3.2.2. Three-Dimensional Training and Validation Data Slicing

Three-dimensional training data were generated from the seven synthetic volumes. The input size for the 3D network(s) was set as 64 × 64 × 64 voxels (sub-volumes). The slicing was performed with a stride = 56, consistent in all x, y, and z directions, resulting in 7 × 9^3^ = 5103 (64 × 64 × 64) 3D pair images (data and respective labels). From these, 4465 random pairs (87.5%) were used as training data and the remaining 638 pairs (12.5%) as validation data (see Table 1). Lastly, the previously discussed augmentations were applied in a random fashion to each sub-volume and not to the seven synthetic volumes as a whole. This strategy increased the number of combinations of the augmentations and, therefore, improved generalization (random augmentations were applied to more samples.)

#### 3.2.3. Neural Network Architectures and Training Parameters

**Figure 5:**
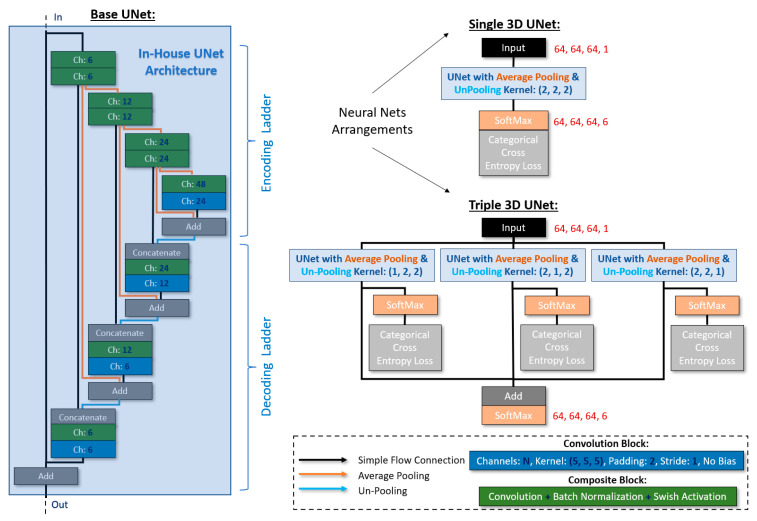
The proposed Single_UNet and Triple_UNet architectures.

As stated previously, one of the goals of the DCNNs was to alleviate the complication of problematic semantic segmentation of small or thin components with current state-of-the-art architectures. The proposed 3D architectures were inspired by the well-established U-Net/V-Net architectures [4,20]. We set the input size as 64, 64, 64, 1_channel, and we used only a few channels in the consecutive convolutions in order to avoid overfitting and achieve better generalization. The selected non-linear activation function was Swish [29] (for its continuity and superiority in this context compared to ReLU), and we employed batch normalization blocks always before the activation functions (except the final one at the output). Furthermore, the input of each step in the network’s encoding ladder was added to the output of the equivalent step in the decoding ladder. Then, the output (sum) of each of these skip connection blocks was concatenated with the output of the equivalent step from the encoding ladder before being fed into the next step of the decoding ladder. The final (at the output) activation function was SoftMax, and the chosen loss function was categorical cross-entropy with 6 classes (voids, fibers, IMs, eutectic Si, SiC particles, and Al matrix: 0 to 5). Finally, we employed average pooling and un-pooling functions for the downscaling and upscaling, respectively. We devised two arrangements. In the first one, we followed the conventional kernels for average pooling and un-pooling: kernel size = (2, 2, 2). This arrangement will be referred to as <Single_UNet> from this point forth. In the second arrangement, we structured a composite neural network consisting of three independent U-Nets, each bearing its own SoftMax and loss function during training. The kernel sizes for average pooling and un-pooling were (1, 2, 2), (2, 1, 2), and (2, 2, 1) for each sub-net, respectively. During forward passing, the individual SoftMax functions were eliminated, and the respective outputs were added together before being fed into a new SoftMax function. This arrangement will be referred to as <Triple_UNet> from this point forth. A detailed sketch of the proposed architectures is illustrated in Figure 5.

The networks described above were designed and trained with Sony’s Neural Network Libraries [30] on a workstation equipped with a GeForce RTX 3090 (Nvidia, Santa Clara, CA, USA), an Intel Pentium i7 CPU (Intel, Santa Clara, CA, USA), and 32 GB of memory. The ADAM algorithm [31] was chosen as the optimizer (parameters: initial learning rate/alpha = 10^−4^, beta1 = 0.9, beta2 = 0.999, updated every iteration), and the selected input batch size was 48. The learning rate was updated exponentially on every epoch with a learning rate multiplier of LRM = 0.9. Moreover, a random shuffling strategy was adopted for the training dataset on each epoch. Finally, the maximum number of epochs was set as 50. Both training and validation errors were recorded, but the final learnable parameters were taken from the epoch that minimized the validation error.

#### 3.2.4. Forwarding Strategies

**Figure 6:**
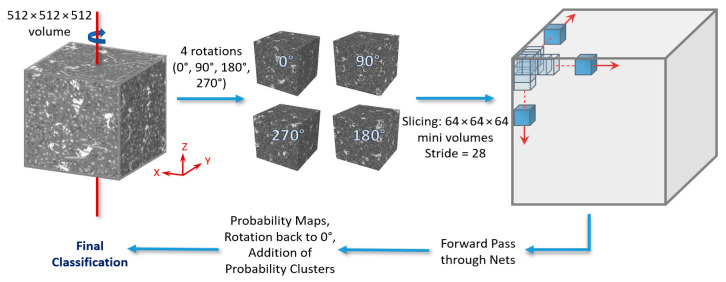
The MultiView forwarding strategy (for a detailed description, please refer to the text).

The 512 × 512 × 512 testing volumes fed into the networks were sliced with a stride = 28, consistent in all x, y, and z directions. This was smaller than the stride used for the training/validation data slicing for increased accuracy. Thus, for 6 classes, 4913 × 6 = 29,478 probability maps (per testing volume) were the output from the networks, which were then reformed with the same stride into 6 probability volumes (one for each class). For each voxel, a class was assigned (0 to 5) based on the highest probability from each class volume, resulting in the final segmentation volume. This slicing/reformation method will be referred to as <SingleView> forwarding strategy from this point forth. Moreover, a second forwarding strategy was examined. It consisted of essentially the same strategy as the above, but with the initial volume rotated four times around the z-axis (0°, 90°, 180°, 270°) before being sliced (in the same manner as before). The resulting 4 × 6 = 24 probability volumes were then rotated back to 0° and added together (in class clusters of 4) before the final classification. This will be referred to as the <MultiView> forwarding strategy (see Figure 6) from this point forth.

## 4. Application: Results and Discussion

**Figure 7:**
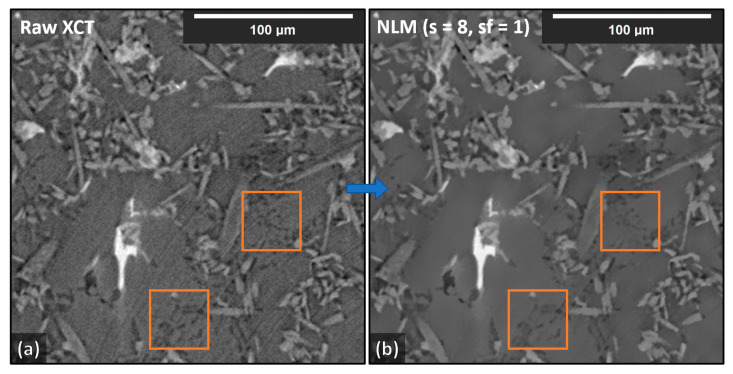
AlSi12CuMgNi MMC XCT reconstruction cross-sections (512 × 512, XY-plane view) before (unconditioned) and after (conditioned) the application of an NLM Filter with s = 8 and sf = 1. The squares show regions where some structural loss (due to filtering) can be observed. (**a**) without filtering, (**b**) with NLM filtering.

Four 512 × 512 × 512 volumes were randomly cut from a larger 1800 × 1800 × 800 XCT reconstructed volume of the AlSi12CuMgNi MMC. Although the XCT volumes were of rather good quality in terms of reconstruction clarity with only some artifacts, we further conditioned them (after the reconstruction) with the application of a non-local-means (NLM) filter [11,32,33], with Sigma = 8 and Smoothing_Factor = 1. This significantly reduced the image noise. Nevertheless, some blurring still remained in the conditioned volumes. Furthermore, some image structural loss was observed on the smallest objects because of the filtering; such loss of details was considered acceptable. In Figure 7, an XCT reconstruction (with a standard filtered back-projection reconstruction algorithm, see Ref. [11]) cross-section is shown before and after the application of the filter.

For each of the four experimental XCT reconstructed volumes, a random cross-section normal to the z-axis was selected and cut. Afterward, the resulting four slices were manually labeled, annotating the existing six microstructural phases. The performance was assessed based on both the eighth synthetic MMC volume (reserved for testing) and on the four experimental XCT volumes (average result of the four labeled slices). More specifically, based on the proposed neural network architectures and forwarding strategies, the following cases were examined:

1. No data augmentations + Single_UNet + SingleView;
2. Data augmentations + Single_UNet + SingleView;
3. Data augmentations + Single_UNet + MultiView;
4. Data augmentations + Triple_UNet + MultiView.

The performance of the latter combinations was quantitively assessed with the Dice coefficient as in [11], commonly employed for segmentation performance assessment of CNNs in semantic segmentation tasks. Dice is a precision metric that rewards correctly segmented pixels and penalizes the incorrect ones altogether and is defined as:

Dice = 2TP/(2TP + FP + FN) (1)

where TP, FP, and FN are the numbers of true-positive, false-positive, and false-negative pixels/voxels, respectively.

**Figure 8:**
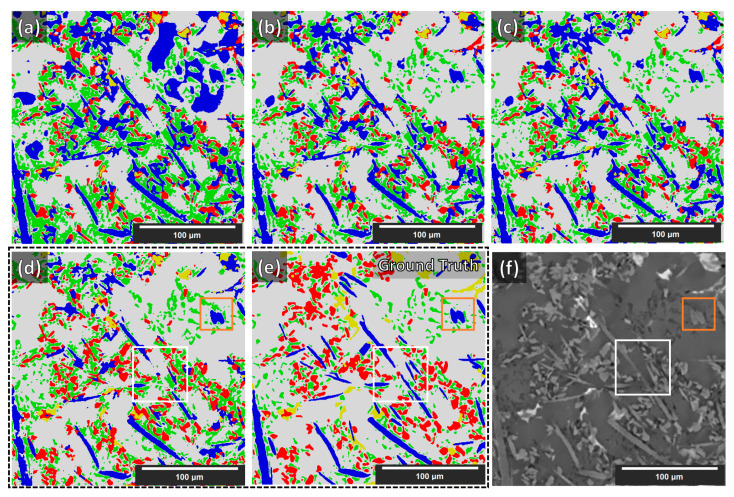
Segmentation results of the experimental NLM-conditioned AlSi12CuMgNi MMC XCT data for all examined cases. Images (**a**–**d**): cases 1–4 above, respectively. Images (**e**,**f**): manual labels (ground truth) and the respective slice from the XCT reconstruction volume, respectively. Labels → blue—fibers; yellow—IMs; green—Si; red—SiC particles; gray—Al matrix. The white and orange squares are regions of interest described in the text below.

**Figure 9:**
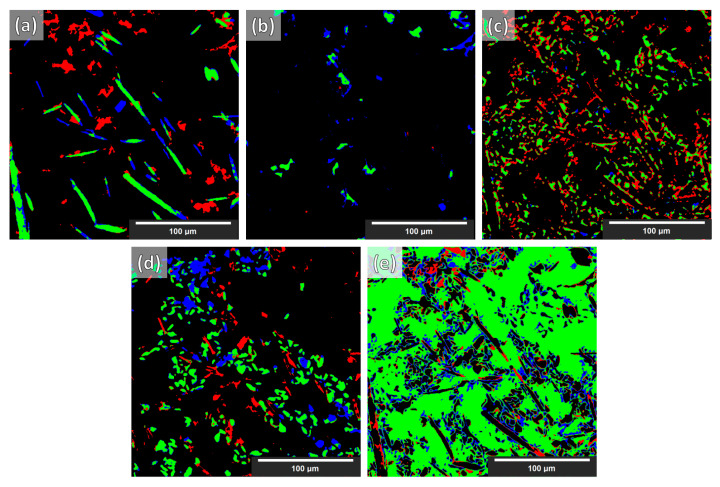
TP, FP, and FN error maps of the segmentation for case 4 (i.e., data augmentations + Triple_UNet + MultiView) based on NLM-conditioned experimental data. Images → (**a**) fibers; (**b**) IMs; (**c**) Si; (**d**) SiC particles; (**e**) Al matrix. Labels → red—FP; blue—FN; green—TP.

**Figure 10:**
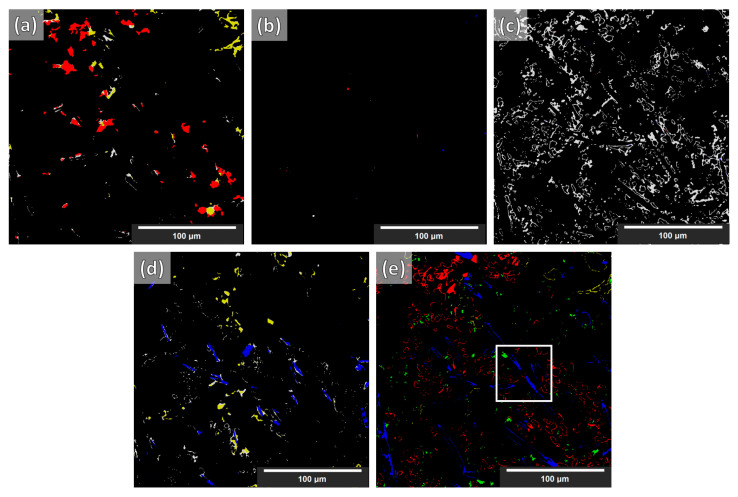
FP maps with labels of the segmentation for case 4 (i.e., data augmentations + Triple_UNet + MultiView) based on NLM-conditioned experimental data. Images → (**a**) fibers; (**b**) IMs; (**c**) Si; (**d**) SiC particles; (**e**) Al matrix. Labels → blue—fibers; yellow—IMs; green—Si; red—SiC particles; gray—Al matrix. (The colored labels indicate what the false-positive regions in each phase should have been if they had been correctly segmented.) The white square is a region of interest described in the text below.

As stated in [11], with the current stage of deep learning development, any score greater than 0.7 is considered an acceptable segmentation result (Dice = 0 indicates no overlap, Dice = 1 indicates perfect match). The Dice coefficient from the segmentation of the synthetic volume, of the NLM-conditioned experimental XCT volume, and finally of the un-conditioned experimental CT volume (for the examined cases) are given in Table 2. In Figure 8, a slice from the NLM-conditioned XCT data segmentation result is shown for all examined cases. In Figure 9, the TP, FP, and FN maps are illustrated for all microstructural phases for the same slice as for Figure 8, but for case four only (i.e., data augmentations + Triple_UNet + MultiView case). In Figure 10, the FP maps illustrated in Figure 9 were assigned the correct class based on the manually labeled ground-truth slices.

The purpose of this study was to render a DCNN capable of generalized learning from synthetic XCT data and to project this knowledge into semantic segmentations capable of reproducing as correctly as possible experimental XCT data. The sensitivity and performance of the four examined cases were initially assessed on the segmentation of a synthetic volume reserved for this purpose. As reported in Table 2, for case one (i.e., plain, Single_UNet, SingleView), the Dice coefficient is 0.99, i.e., the segmentation is almost perfect. This is consistent across all microstructural phases. These scores validate the accuracy of the proposed base UNet architecture, despite the relatively small number of channels employed in the convolutional layers. In case two (i.e., augmentation, Single_UNet, SingleView), the introduction of the augmentations has a negative impact on the accuracy of the synthetic XCT data segmentation, as the drop in the Dice coefficients indicates. This is reasonable since the initial synthetic data (before the augmentations) incorporate only a few grayscales. Thus, without any augmentations, the UNet performs better as fewer patterns have to be learned. Typically, augmentations are employed when the training data are not sufficient in numbers (to create more). However, when data are sufficient, no augmentation is required as it would introduce an unnecessary generalization and therefore decrease the performance. On the other hand, the multiple view approach in case three (i.e., data augmentations + Single_UNet + MultiView) has a positive impact on the performance when segmenting the synthetic data. In case four (i.e., data augmentations + Triple_UNet + MultiView), the introduction of the triple UNet arrangement has an adverse effect with respect to case three, reducing the Dice coefficient again. It is to be noted that despite the slight decrease, the lowest overall Dice coefficient achieved is 0.98, and the lowest individual phase Dice coefficient is 0.93. Thus, the validity of the proposed cases is confirmed.

Although increasing sophistication of the data treatment (cases one to four) reduces the performance for the segmentation of synthetic XCT data, in the case of the conditioned experimental XCT datasets, this path leads to a progressively increasing performance according to the Dice coefficients in Table 2. The application of the suggested augmentations during training increases the overall Dice coefficient from 0.62 to 0.73 (cases one to two, respectively). This is visually reflected when comparing the segmentation maps in Figure 8a,b with the ground truth (manual segmentation) in Figure 8e. It can clearly be seen that the large (blue) regions of the Al matrix that were previously misinterpreted as fibers are correctly segmented in case two. The same applies to the overexpanded (green) Si phase: much of it was initially wrongly segmented (instead of being correctly labeled as Al matrix). A better distinction between SiC particles and alumina fibers is visible as well in case two. All such improvements mean a considerable increase in the individual phase Dice coefficients. Contrarily, a decrease in the Dice coefficient of the IMs phase segmentation (yellow) is observed, although this is not visible in the segmentation map of Figure 8b. As with the synthetic data segmentation, in case three, the multiple view strategy is beneficial to increase the overall Dice coefficient, but the IMs individual Dice coefficient remains unchanged. A meaningful increase in precision was observed for the SiC particles. According to the other individual Dice coefficients, this can be attributed to a slightly better distinction between alumina fibers and SiC particles, and to a lesser extent, to the better geometrical identification of the SiC particles/Al matrix interface. These aspects are not easily visible by just examining the segmentation maps in Figure 8b,c. The situation is different for the transition between cases three and four. Clearly, in Figure 8d, the classification between the rival alumina fibers and SiC particles improves considerably, with more regions being correctly labeled as SiC particles instead of fibers and vice versa. The same happens between the SiC particles and IMs phases: fewer IMs are mislabeled as particles. This is equally reflected in the increase in the Dice coefficients for the fibers, the SiC particles, and the IMs, as shown in Table 2. Furthermore, the clustering of the Si phase is reduced even further, although this is not immediately visible in the segmentation images. The IM segmentation, on the other hand, is distinguishably closer to the manually annotated ground truth in Figure 8e. This is reflected in the increase in its Dice coefficient, reaching its highest precision. The final overall Dice coefficient of 0.77 in the most complex case, case four, is above the 0.70 threshold to consider the segmentation successful. Such overall Dice coefficient is regarded acceptable considering the complexity of the problem, in spite of the fact that the fiber and IM phases segmentations achieve relatively low Dice coefficients (0.48 and 0.55, respectively). As the error map of the fibers in Figure 9a unveils, there are significant FP and FN regions contributing towards this performance. The FP regions are mostly due to mislabeled fibers (as SiC particles), according to Figure 10a, while the FN regions (shown in Figure 9a in blue) are mostly scattered in Figure 10d,e (in blue). Interestingly, the fibers and SiC particle segmentation maps are almost negatives of each other. The IMs in Figure 9b have no FP regions, and their FN regions are shared between fibers and SiC particles. The confusion between fibers, SiC particles, and IMs was expected because of their similar grayscales (and sometimes shapes). For the Si phase, all FP regions shown in Figure 9c should be Al matrix regions instead, according to Figure 10c. Interestingly, the few FN regions (in Figure 9c) are mislabeled as Al matrix regions, as Figure 10e indicates. Overall, it can be deduced that the Si phase is “over-segmented”, with the segmented Si boundaries expanding within the Al matrix if we refer to the ground-truth labels (manually annotated). There is no guarantee that the manual annotations are 100% accurate as these are prone to human error. In fact, a closer inspection of Figure 8f shows that there is not always a clear distinction between microstructural phase boundaries (orange square: blurry Si/Al matrix boundary in Figure 8d–f). The phase boundary uncertainty can be seen more clearly in Figure 10e for almost every phase/Al matrix boundary. Similarly, the wrongly mislabeled fiber tips (as Al matrix) can be again attributed to the blurry fibers/Al matrix interface (white square in Figure 8d–f and Figure 10f).

**Figure 11:**
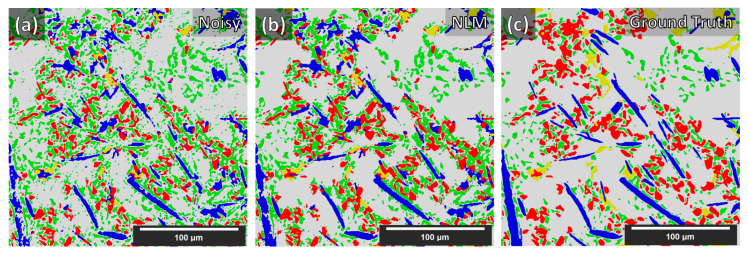
Segmentation maps comparison of the unconditioned (noisy) and NLM-conditioned experimental data for case 4 (i.e., data augmentations + Triple_UNet + MultiView.) Segmentation maps: (**a**) Unconditioned experimental data, (**b**) NLM-conditioned experimental data, (**c**) Ground Truth.

We believe that existing artifacts within the XCT experimental data were a major hurdle for achieving generalized segmentation based on training from synthetic data. With manual annotation of the training data, these artifacts are included in the training process and therefore are learned by the DCNN. Ideally, XCT data must be as clean (no noise) and as sharp (no blur) as possible to achieve even higher segmentation quality with synthetic data training. The latter requirement becomes important for more complex microstructures containing phases with similar XCT grayscales, such as the Al–Si MMC presented in this study. This is clearly demonstrated in Table 2 with the Dice coefficient achieved for the unconditioned (noisy) data (shown in Figure 7a) and in Figure 11. Despite the unconditioned XCT data being of good quality by modern standards, the low noise and blur present have an adverse effect on the Dice coefficients for every phase. The application of the NLM filter to the reconstructed XCT data improved the segmentation (Dice) score considerably. However, more sophisticated XCT data conditioning methods, such as in [34,35,36], could potentially further improve the result. It should be reported that the equivalent 2D approach results in worse precision. Moreover, a change from 64 × 64 × 64 input to 96 × 96 × 96 input size for the 3D case provided no increase in performance. Finally, we report that we attempted double and quadruple UNet arrangements (the quadruple arrangement was essentially a combination of the Single_UNet and the Triple_UNet); however, the triple arrangement achieved the best generalization with synthetic data training.

## 5. Conclusions and Outlook

We propose a complete and new strategy to avoid manually annotating training data for the segmentation of X-ray tomography data and increase the quality of such segmentation. Our strategy is based on the generation of synthetic training (and validation) data and special deep convolutional neural networks (DCNNs) for the augmentation of such data (in particular, the experimental data).

(A) The method developed for generating synthetic XCT data for materials with complex microstructures, (B) the proposed conversion approach of these data into relevant training data (certain intensity of contrast, brightness, noise, and blur augmentations), (C) the proposed novel 3D DCNN architecture (Triple_UNet), and (D) the proposed forwarding strategy (MultiView forwarding strategy), are appropriate to achieve our final goal, based on the overall Dice coefficient achieved (on both synthetic and experimental test data). We show that high automatic segmentation precision can be achieved on artifact-free XCT data, not only overall but also for each individual phase. As work for the future, it is worth exploring whether more advanced XCT data conditioning methods (compared to the NLM filter) can enhance the segmentation precision. The significance of this work lies in the effectiveness of the proposed methods using truly automatic segmentation of XCT data with neural networks, i.e., without resorting to time-consuming manual annotation. This approach will further foster the use of AI in material science analysis and accelerate scientific output since our method should be easily applicable to other materials and imaging techniques.

## Figures and Tables

**Table 1:** Training and validation data summary.

Synthetic Al-Si MMC CT Volumes Fabricated	Volumes Used for Training/Validation Data (Random Selection)	Volumes Reserved for Testing	Training/Validation Volumes Slicing Stride	Total Sub-Volume Pairs	Training Pairs	Validation Pairs
8	7	1	56	5103	4465	638

**Table 2:** Quantitative assessment of the synthetic XCT segmentation, NLM-filtered segmented experimental XCT data, and the un-conditioned segmented experimental XCT data for all cases (voids phase is not included as it was absent in the ground-truth slices).

(Case)	Al_2_O_3_ Fibers	IMs	Si	SiC Particles	Al Matrix		Overall
**Synthetic Data—DICE**							
**(1) Plain, Single Unet, Single View**	0.99	0.99	0.97	0.99	0.99		**0.99**
**(2) Augmentation, Single Unet, Single View**	0.97	0.98	0.93	0.96	0.98		**0.98**
**(3) Augmentation, Single Unet, Multi View**	0.98	0.99	0.94	0.97	0.99		**0.98**
**(4) Augmentation, Triple Unet, Multi View**	0.97	0.98	0.93	0.97	0.98		**0.98**
							
**NLM8 Conditioned Experimental Data—DICE**							
**(1) Plain, Single Unet, Single View**	0.34	0.53	0.50	0.48	0.76		**0.62**
**(2) Augmentation, Single Unet, Single View**	0.45	0.48	0.58	0.54	0.86		**0.73**
**(3) Augmentation, Single Unet, Multi View**	0.46	0.48	0.59	0.59	0.87		**0.74**
(**4) Augmentation, Triple Unet, Multi View**	0.49	0.55	0.60	0.66	0.87		**0.77**
							
**Not Conditioned Experimental Data—DICE**							
**(4) Augmentation, Triple Unet, Multi View**	0.44	0.42	0.55	0.58	0.84		**0.72**

## Data Availability

The data that support the findings of this study are available from the corresponding author, AT, upon reasonable request.

## Appendix A

This appendix contains details on the synthetic microstructures’ statistics:

**Table A1:** Synthetic Al–Si MMCs 1–4 specifications.

**Synthetic AlSi MMC 1**															
	Repository	Positioning Function	Size_X	Size_Y	Size_Z	Vol. Fraction	Positioning	Rotation X-Axis	Rotation Z-Axis	Resize_X	Resize_Y	Resize_Z	Gray Value	Local Contrast	Priority
Fibers (F)	FS 1	HVF	512	512	512	5	Random	Normal with STD = 6.5	Random	-	-	-	93	+/− 10%, t = 4 voxels	1
Intermetallics (M)	IM	SVF	512	512	512	5	Random	Random	Random	64–128	64–128	64–128	214	-	2
Si (S)	CHX 2	SVF	512	512	512	16	Random	Random	Random	15–45	45,056	15–45	70	-	3
SiC Particles (P)	CHX 1	HVDF, Dep: F	512	512	512	8	Random	Random	Random	10–35	10–35	10–35	129	-	1
Voids (V)	CHX 1	SVDF, Dep: F,P	512	512	512	0.1	Random	Random	Random	45,163	45,163	45,163	0	-	4
Al Matrix (A)	-	-	-	-	-	-	-	-	-	-	-	-	91	-	5
															
**Synthetic AlSi MMC 2**															
	Repository	Positioning Function	Size_X	Size_Y	Size_Z	Vol. Fraction	Positioning	Rotation X-Axis	Rotation Z-Axis	Resize_X	Resize_Y	Resize_Z	Gray Value	Local Contrast	Priority
Fibers (F)	FS 2	HVF	512	512	512	5	Random	Normal with STD = 6.5	Random	-	-	-	98	+/− 10%, t = 3 voxels	1
Intermetallics (M)	IM	SVF	512	512	512	5	Random	Random	Random	64–128	64–128	64–128	214	-	2
Si (S)	CHX 2	SVF	512	512	512	14	Random	Random	Random	15–45	45,056	15–45	72	-	3
SiC Particles (P)	CHX 1	HVDF, Dep: F	512	512	512	8	Random	Random	Random	10–35	10–35	10–35	136	-	1
Voids (V)	CHX 1	SVDF, Dep: F,P	512	512	512	0.1	Random	Random	Random	45,163	45,163	45,163	0	-	4
Al Matrix (A)	-	-	-	-	-	-	-	-	-	-	-	-	90	-	5
															
**Synthetic AlSi MMC 3**															
	Repository	Positioning Function	Size_X	Size_Y	Size_Z	Vol. Fraction	Positioning	Rotation X-Axis	Rotation Z-Axis	Resize_X	Resize_Y	Resize_Z	Gray Value	Local Contrast	Priority
Fibers (F)	FS 3	HVF	512	512	512	5	Random	Normal with STD = 6.5	Random	-	-	-	125	-	1
Intermetallics (M)	IM	SVF	512	512	512	5	Random	Random	Random	64–128	64–128	64–128	208	-	2
Si (S)	CHX 2	SVF	512	512	512	15	Random	Random	Random	15–45	45,056	15–45	68	-	3
SiC Particles (P)	CHX 1	HVDF, Dep: F	512	512	512	8	Random	Random	Random	10–35	10–35	10–35	129	-	1
Voids (V)	CHX 1	SVDF, Dep: F,P	512	512	512	0.1	Random	Random	Random	45,163	45,163	45,163	0	-	4
Al Matrix (A)	-	-	-	-	-	-	-	-	-	-	-	-	88	-	5
															
**Synthetic AlSi MMC 4**															
	Repository	Positioning Function	Size_X	Size_Y	Size_Z	Vol. Fraction	Positioning	Rotation X-Axis	Rotation Z-Axis	Resize_X	Resize_Y	Resize_Z	Gray Value	Local Contrast	Priority
Fibers (F)	FS 4	HVF	512	512	512	5	Random	Normal with STD = 6.5	Random	-	-	-	139	-	1
Intermetallics (M)	IM	SVF	512	512	512	5	Random	Random	Random	64–128	64–128	64–128	212	-	2
Si (S)	CHX 2	SVF	512	512	512	15	Random	Random	Random	15–45	45,056	15–45	70	-	3
SiC Particles (P)	CHX 1	HVDF, Dep: F	512	512	512	8	Random	Random	Random	10–35	10–35	10–35	137	-	1
Voids (V)	CHX 1	SVDF, Dep: F,P	512	512	512	0.1	Random	Random	Random	45,163	45,163	45,163	0	-	4
Al Matrix (A)	-	-	-	-	-	-	-	-	-	-	-	-	91	-	5

**Table A2:** Synthetic Al–Si MMCs 5–8 specifications.

**Synthetic AlSi MMC 5**															
	Repository	Positioning Function	Size_X	Size_Y	Size_Z	Vol. Fraction	Positioning	Rotation X-Axis	Rotation Z-Axis	Resize_X	Resize_Y	Resize_Z	Gray Value	Local Contrast	Priority
Fibers (F)	FS 5	HVF	512	512	512	5	Random	Normal with STD = 6.5	Random	-	-	-	120	-	1
Intermetallics (M)	IM	SVF	512	512	512	5	Random	Random	Random	64–128	64–128	64–128	214	-	2
Si (S)	CHX 2	SVF	512	512	512	14	Random	Random	Random	15–45	45,056	15–45	74	-	3
SiC Particles (P)	CHX 1	HVDF, Dep: F	512	512	512	7	Random	Random	Random	10–35	10–35	10–35	137	-	1
Voids (V)	CHX 1	SVDF, Dep: F,P	512	512	512	0.1	Random	Random	Random	45,163	45,163	45,163	0	-	4
Al Matrix (A)	-	-	-	-	-	-	-	-	-	-	-	-	95	-	5
															
**Synthetic AlSi MMC 6**															
	Repository	Positioning Function	Size_X	Size_Y	Size_Z	Vol. Fraction	Positioning	Rotation X-Axis	Rotation Z-Axis	Resize_X	Resize_Y	Resize_Z	Gray Value	Local Contrast	Priority
Fibers (F)	FS 6	HVF	512	512	512	2	Random	Normal with STD = 6.5	Random	-	-	-	106	+/− 10%, t = 2 voxels	1
Intermetallics (M)	IM	SVF	512	512	512	1	Random	Random	Random	64–128	64–128	64–128	214	-	2
Si (S)	CHX 2	SVF	512	512	512	1	Random	Random	Random	15–45	45,056	15–45	71	-	3
SiC Particles (P)	CHX 1	HVDF, Dep: F	512	512	512	1	Random	Random	Random	10–35	10–35	10–35	126	-	1
Voids (V)	CHX 1	SVDF, Dep: F,P	512	512	512	0.3	Random	Random	Random	45,163	45,163	45,163	0	-	4
Al Matrix (A)	-	-	-	-	-	-	-	-	-	-	-	-	91	-	5
															
**Synthetic AlSi MMC 7**															
	Repository	Positioning Function	Size_X	Size_Y	Size_Z	Vol. Fraction	Positioning	Rotation X-Axis	Rotation Z-Axis	Resize_X	Resize_Y	Resize_Z	Gray Value	Local Contrast	Priority
Fibers (F)	FS 7	HVF	512	512	512	3	Random	Normal with STD = 8.5	Random	-	-	-	145	-	1
Intermetallics (M)	IM	SVF	512	512	512	0.5	Random	Random	Random	64–128	64–128	64–128	208	-	2
Si (S)	CHX 2	SVF	512	512	512	0.5	Random	Random	Random	15–45	45056	15–45	76	-	3
SiC Particles (P)	CHX 1	HVDF, Dep: F	512	512	512	0.5	Random	Random	Random	10–35	10–35	10–35	131	-	1
Voids (V)	CHX 1	SVDF, Dep: F,P	512	512	512	0.1	Random	Random	Random	45,163	45,163	45,163	0	-	4
Al Matrix (A)	-	-	-	-	-	-	-	-	-	-	-	-	93	-	5
															
**Synthetic AlSi MMC 8**															
	Repository	Positioning Function	Size_X	Size_Y	Size_Z	Vol. Fraction	Positioning	Rotation X-Axis	Rotation Z-Axis	Resize_X	Resize_Y	Resize_Z	Gray Value	Local Contrast	Priority
Fibers (F)	FS 8	HVF	512	512	512	5	Random	Normal with STD = 6.5	Random	-	-	-	98	+/− 10%, t = 3 voxels	1
Intermetallics (M)	IM	SVF	512	512	512	5	Random	Random	Random	64–128	64–128	64–128	214	-	2
Si (S)	CHX 2	SVF	512	512	512	12	Random	Random	Random	15–45	45056	15–45	70	-	3
SiC Particles (P)	CHX 1	HVDF, Dep: F	512	512	512	8	Random	Random	Random	10–35	10–35	10–35	136	-	1
Voids (V)	CHX 1	SVDF, Dep: F,P	512	512	512	0.1	Random	Random	Random	45,163	45,163	45,163	0	-	4
Al Matrix (A)	-	-	-	-	-	-	-	-	-	-	-	-	91	-	5

**Table A3:** Particles and fibers repositories specifications.

	No.	Size_X	Size_Y	Size_Z	Generation Limit	Starvation Limit	Iterations	Initial Voxels Alive
**Cellular Automata Masks (CA)**	110	128	128	128	14	13	5	50–55%
								
	No.	Size_X	Size_Y	Size_Z	No. Points	Alpha Rad.		
**Concave Particles (CCP)**	125	128	128	128	20–50	0.6–0.4		
								
	No.	Method						
**Intermetallics (IM)**	190	Random Intersections of CA & CCP						
								
	No.	Size_X	Size_Y	Size_Z	No. Points			
**Convex Particles (CXH 1)**	160	64	64	64	45,229			
								
	No.	Size_X	Size_Y	Size_Z	No. Points			
**Convex Particles (CXH 2)**	130	64	8	64	10–60			
								
		(Y-Axis)						
	No.	Length	Rad.					
**Fibers (FS 1)**	150	30–380	20–35					
**Fibers (FS 2)**	150	30–380	45,219					
**Fibers (FS 3)**	150	30–380	45,153					
**Fibers (FS 4)**	150	30–380	45,000					
**Fibers (FS 5)**	150	30–500	45,219					
**Fibers (FS 6)**	150	30–500	45,163					
**Fibers (FS 7)**	150	30–500	44,993					
**Fibers (FS 8)**	150	30–320	45,160					
